# The 3D landscape of infection: spatial organization and *in vitro* models

**DOI:** 10.3389/fimmu.2026.1898257

**Published:** 2026-08-24

**Authors:** Oliver Goldmann, Eva Medina

**Affiliations:** Infection Immunology Research Group, Helmholtz Centre for Infection Research, Braunschweig, Germany

**Keywords:** abscess, biofilm, bioprinting, bioreactor, granuloma, organoids, organ-on-chip, spheroids

## Abstract

Microbial infections rarely consist of free-floating cells. Instead, pathogens and immune cells are typically organized into complex three-dimensional (3D) structures, including biofilms, abscesses, and granulomas, which can serve to constrain pathogen dissemination. At the same time, these architectures create protective microenvironments that promote microbial persistence, immune evasion, and increased resistance to antimicrobial therapies. A mechanistic understanding of how spatially organized tissue structures and local microenvironmental cues shape host-pathogen dynamics may open new avenues for therapeutic interventions, for example by improving targeted drug delivery, enhancing antibiotic penetration, or disrupting protective microbial niches. In this review, we summarize emerging insights into the 3D landscape of infections and highlight advances in experimental platforms that more faithfully recapitulate tissue architecture, including 3D cell culture systems, bioprinted tissue, organoids, and organ-on-chip technologies. Finally, we discuss how integrating spatial parameters into infection models reshapes our understanding of pathogenesis and opens new conceptual and therapeutic avenues for combating infections.

## 1 Introduction

The concept of the “3D in infection” refers to the fact that infections occur and develop within the complex 3D structure of living tissues. In the human body, pathogens such as bacteria, viruses, and fungi spread through layered tissues, extracellular matrices, and interconnected systems such as blood vessels and airways. The immune response is likewise shaped by these 3D structures. Immune cells migrate through tissues in response to chemokine gradients, and their activity is further influenced by local conditions such as oxygen levels and signaling molecules (1, 2). At epithelial barriers, immune defense is initiated through the coordinated action of epithelial cells, resident immune cells, and antimicrobial mediators that detect and respond to invading pathogens (3). Beyond the epithelial surface, vascular and lymphatic compartments support immune surveillance by enabling the recruitment of circulating immune cells, the transport of soluble mediators, and the drainage of antigens and inflammatory signals to lymphoid tissues (4). Pathogens may exploit this spatial complexity to evade immune clearance, for example by occupying protected niches, disrupting chemokine signaling, or altering tissue architecture (5). Together, these processes underscore the importance of spatial tissue organization in determining the outcome of host-pathogen interactions.

Spatial organization also contributes to the efficacy of therapeutic treatments. Antibiotic penetration within infected tissues can be heterogeneous, resulting in subtherapeutic concentrations in specific regions. Bacteria located in poorly perfused or necrotic areas may be protected from both antimicrobial agents and immune clearance. Similarly, dormant subpopulations in nutrient-limited zones may exhibit increased antibiotic tolerance.

**Figure 1:**
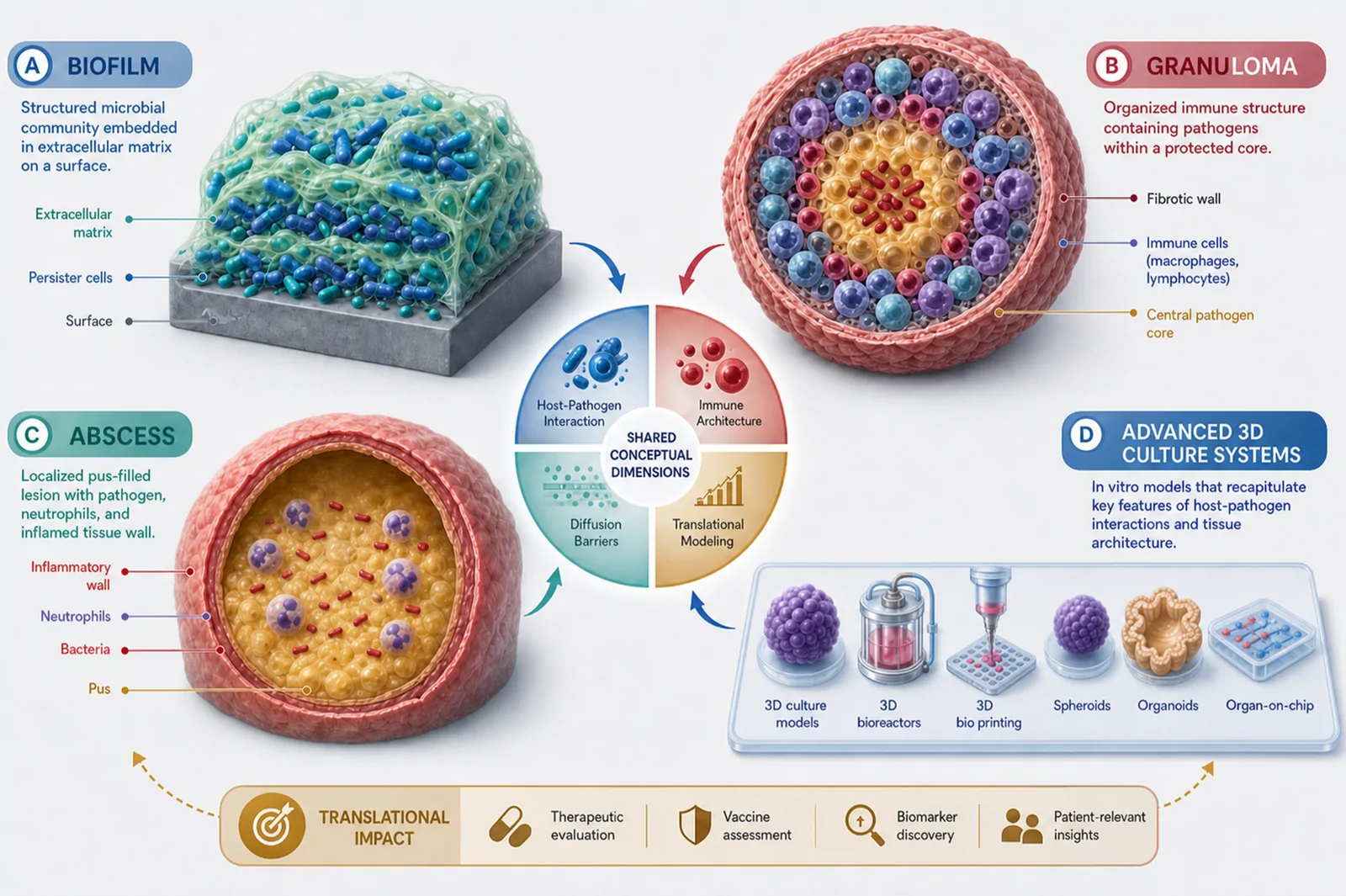
Conceptual illustration of prominent infection-related 3D structures and advanced 3D culture systems discussed in this review. Infection-related 3D structures include **(A)** biofilms, **(B)** granulomas, and **(C)** abscesses. **(D)** Advanced 3D culture systems include 3D culture models, 3D bioreactors, 3D bioprinting, spheroids, organoids, and organ-on-chip systems. This conceptual illustration was generated using AI-based tools.

Among the available models for studying infection, animal models have provided important insights. However, their translational value is frequently limited by inherent physiological differences between species, which may compromise the relevance of experimental findings to humans (6). Likewise, conventional two-dimensional (2D) *in vitro* cell culture systems do not reflect the structural and functional complexity of native tissue and are therefore unable to fully recapitulate the multicellular interactions that underpin disease pathophysiology (7). To address these limitations, substantial progress has been made in recent years in the development of sophisticated 3D *in vitro* systems that more closely mimic the spatial complexity, cellular heterogeneity, and chemical gradients essential for understanding host-pathogen interactions and therapeutic responses (8, 9). These 3D *in vitro* models hold considerable promise for advancing translational research by enabling preclinical drug screening, supporting vaccine design, and facilitating personalized therapeutic approaches, while also helping to reduce reliance on animal models. A summary of the topics addressed in this review is presented in **Figure 1**.

## 2 Spatial organization in infection

Infections are dynamic spatiotemporal processes shaped by the complex 3D architecture of host tissues (10). At sites of infection, pathogens occupy localized microenvironments defined by gradients of oxygen, nutrients, pH, immune mediators, and antimicrobial agents (11). These gradients create distinct ecological niches that strongly influence pathogen behavior. Host immune responses are also spatially organized, with immune cells forming complex structures such as granulomas, abscesses, and biofilm-associated infiltrates. Although these structures can help contain and restrict infection, they may also create protective niches that limit antimicrobial penetration, dampen immune effector functions, and promote pathogen persistence. This spatial heterogeneity drives phenotypic diversity within pathogen populations, supporting metabolic adaptation, immune evasion, and antibiotic tolerance, and thereby influencing disease progression and therapeutic efficacy (10, 11). Representative examples of spatial organization in infection that will be addressed in this review include bacterial biofilms, in which microorganisms form dense, surface-attached communities, as well as granulomas and abscesses, in which immune cells organize to surround and contain pathogens at infection sites. Although the primary function of granulomas and abscesses is to restrict pathogen spread, they can also create protective environments that enable pathogen persistence. A more detailed description of these 3D infection-related structures is provided in the following sections.

### 2.1 Biofilms

A biofilm is a highly organized three-dimensional community of microorganisms attached to a surface and encased in a self-produced extracellular matrix composed of polymers such as proteins, polysaccharides, and nucleic acids (12–14). This matrix provides mechanical strength and stability while modulating the diffusion of molecules throughout the biofilm structure. As a result, gradients in nutrients and other chemical factors, including oxygen, pH, signaling molecules, metabolites, and other solutes, develop across the biofilm. Consequently, distinct microenvironments with varying chemical conditions arise and may be further shaped by the metabolic activity of local microorganisms (15). Microorganisms benefit from this architecture because it facilitates nutrient capture and retention, promotes close cell-cell communication, and enhances collective survival strategies. Importantly, the matrix also acts as a protective barrier, limiting the penetration of antimicrobial agents and shielding resident microbes from the host immune response (16, 17). Consequently, microorganisms within biofilms can tolerate antimicrobial treatment at concentrations far higher than those effective against planktonic cells. As a result, biofilm-associated infections are often difficult to eradicate and may become chronic (18). Biofilms also facilitate horizontal gene transfer between microorganisms, contributing to the spread of antimicrobial resistance genes (19).

**Figure 2:**
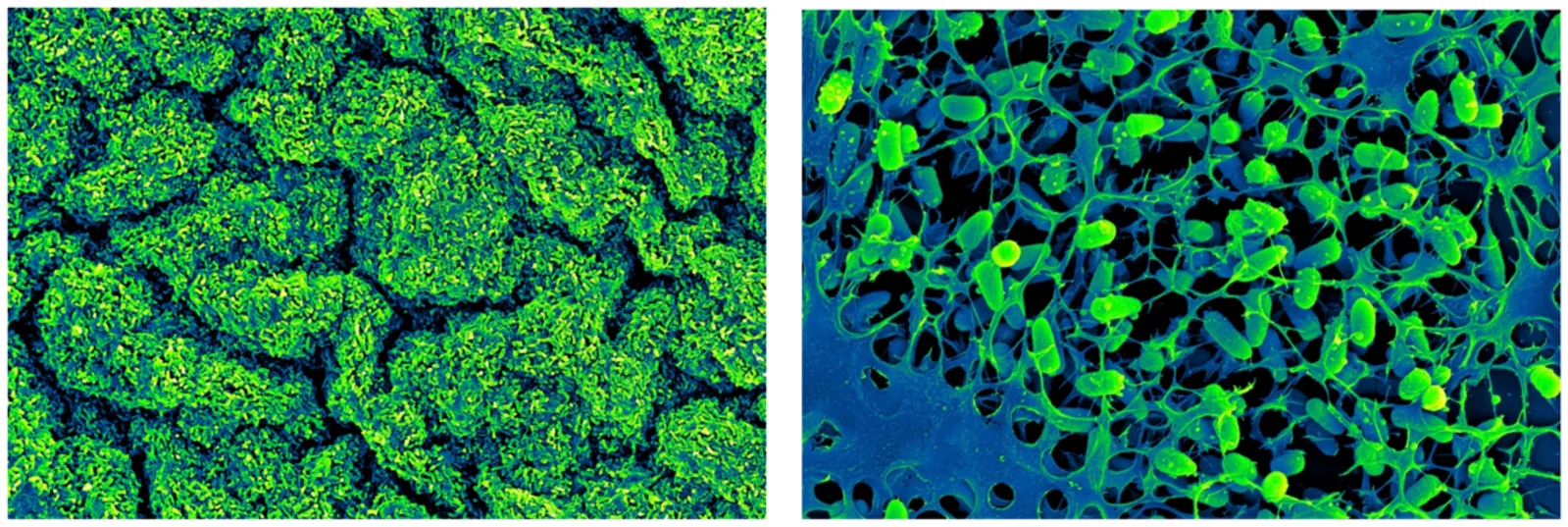
Architecture of *Pseudomonas aeruginosa* biofilms. Artificially colorized scanning electron microscopy images of *P. aeruginosa* biofilms showing dense, highly packed bacterial microcolonies embedded in an extracellular matrix (left) and a looser, more heterogeneous biofilm structure with interconnected matrix networks and dispersed bacteria (right). Bacterial cells are shown in yellow-green, whereas the extracellular polymeric matrix appears blue. Experimental images provided by M. Müsken, ZEIM, HZI, Braunschweig.

A wide range of microorganisms are capable of forming biofilms (20). Among bacterial species, *Pseudomonas aeruginosa* (21) and *Staphylococcus aureus* (22) are among the most commonly implicated. An example of a biofilm produced by *P. aeruginosa* is shown in **Figure 2**.

Bacterial infections in chronic wounds predominantly occur in the form of biofilms (23). Biofilms are also implicated in a wide range of infectious diseases, including periodontitis, cystic fibrosis-associated pneumonia, urinary tract infections, and infections associated with indwelling medical devices (24). Biofilm-associated infections are difficult to treat with standard antibiotics, and the most effective therapeutic strategies typically involve multimodal approaches that combine matrix disruption, interference with signaling pathways, and targeted pathogen killing (25).

Understanding the composition and spatial organization of biofilms is essential for developing more effective strategies for biofilm disruption. A variety of biofilm models have been established (26–29), which have significantly advanced our understanding of the molecular, cellular, and environmental factors that shape biofilm formation and function. Static biofilm models are among the most widely used because they are simple, inexpensive, and easy to handle. They are based on the principle that bacterial adhesion to a surface is a defining feature of biofilms (30). Dynamic biofilm models, in contrast, provide a more advanced setting by incorporating variables such as the controlled flow of liquid across the biofilm surface (31). This makes it possible to study how environmental factors, including shear stress and nutrient supply, influence biofilm structure and behavior. Flow-based systems are often combined with imaging techniques such as confocal laser scanning microscopy, allowing real-time acquisition of detailed 3D representations of biofilm architecture. 3D experimental biofilm models more accurately reflect the structural and functional complexity observed in natural and clinical settings (32–35). Unlike traditional 2D models, 3D systems can capture gradients of nutrients, oxygen, and signaling molecules, as well as the spatial organization of cells. However, several challenges continue to limit their broader application. As biofilm models become more complex to better mimic *in vivo* conditions, they also become more difficult to standardize and reproduce. Variations in materials, growth conditions, and microbial species across laboratories often lead to inconsistent results, highlighting the need for widely accepted protocols. Another major challenge is modeling multi-species biofilms. In nature, biofilms usually consist of diverse microbial communities, but recreating stable and reproducible polymicrobial systems *in vitro* remains technically demanding. Interspecies competition and dynamic shifts in community composition can make these systems difficult to control. In addition, the materials used to construct 3D models, such as hydrogels or synthetic scaffolds, do not always accurately recapitulate the natural extracellular matrix of biofilms. This can affect microbial behavior and reduce physiological relevance. Technical limitations in imaging and analysis also pose important obstacles. Although advanced microscopy techniques allow for visualization of biofilm structures, they are often limited by penetration depth and require complex data processing. Additionally, many 3D biofilm models are also difficult to scale up, making them less suitable for high-throughput applications such as drug screening or antimicrobial testing.

Looking ahead, several promising directions are likely to shape the future of 3D biofilm modeling. Advances in biomaterials are expected to yield more sophisticated and tunable matrices that better replicate natural environments. Improvements in imaging technologies, including light-sheet microscopy and AI-driven analytical tools, will enhance the ability to study biofilm structure and dynamics in greater detail. At the same time, efforts toward standardization will be essential to ensure reproducibility and facilitate comparisons across studies. Expanding the complexity of models to include multispecies communities and even microbiome-level systems will further increase their applicability. Finally, the development of high-throughput and miniaturized platforms, along with the use of patient-derived samples, may enable more personalized approaches to the study of biofilm-associated infections and their treatments. Overall, the future of 3D biofilm models lies in the integration of multiple disciplines to create systems that are both biologically relevant and experimentally practical.

### 2.2 Granulomas

**Figure 3:**
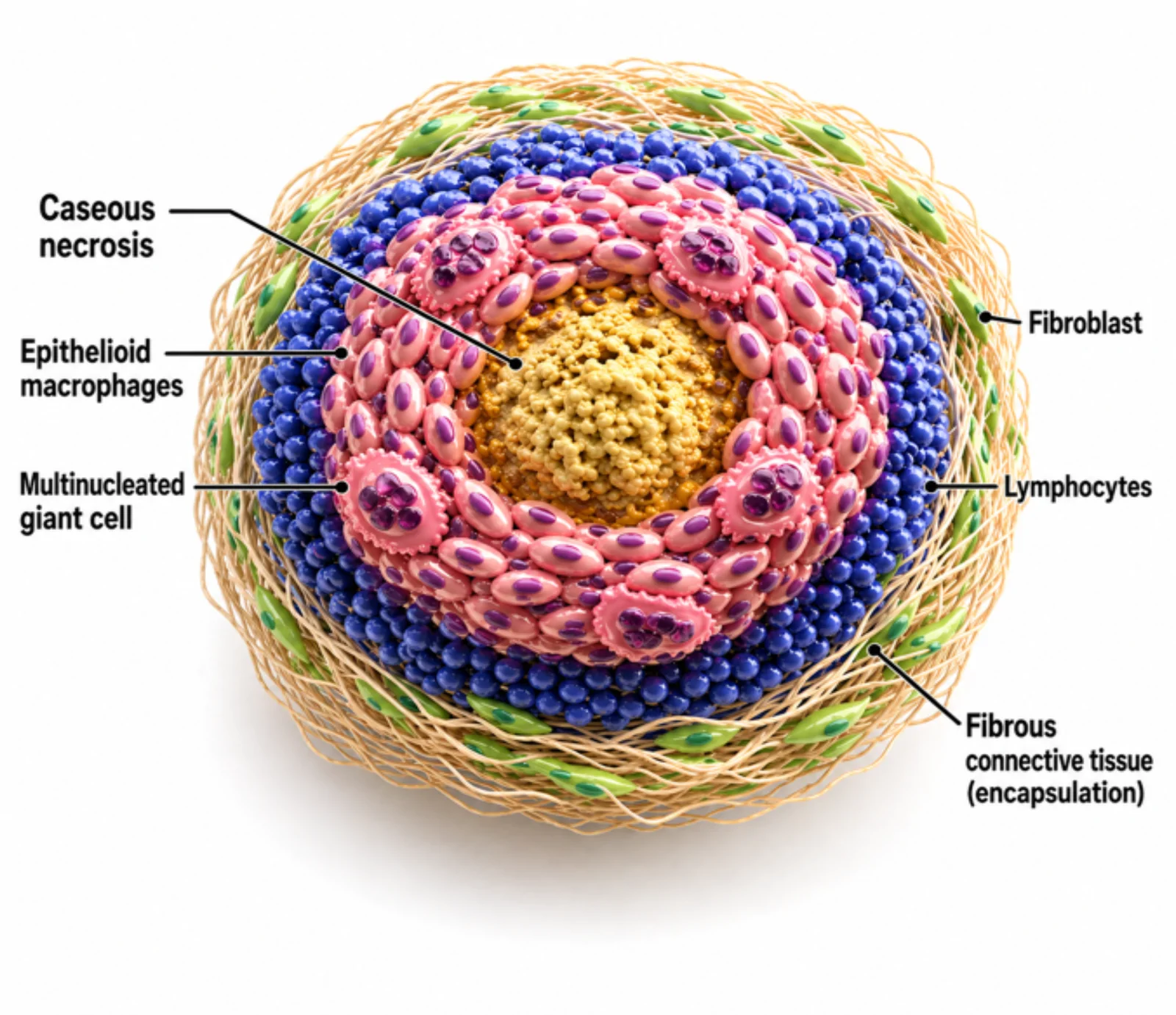
3D schematic representation of a granuloma. A central core of caseous necrosis is surrounded by a layer of epithelioid macrophages, including multinucleated giant cells. This inner zone is encircled by a dense cuff of lymphocytes, reflecting the adaptive immune response. The outer region consists of fibroblasts and collagen fibers, which form a fibrous capsule that partially encapsulates the lesion. This conceptual illustration was generated using AI-based tools.

A granuloma is a chronic inflammatory structure that forms when the immune system attempts to contain certain pathogens such as *Mycobacterium tuberculosis*. Granulomas exhibit a highly organized structure (36). At the center are activated macrophages, which often differentiate into epithelioid cells or fuse to form multinucleated giant cells. These cells play a central role in containing pathogens that resist immediate clearance. In diseases such as tuberculosis, the granuloma core may undergo caseous necrosis, resulting in a characteristic necrotic center associated with persistent infection and ongoing immune-mediated tissue damage (37). This core is surrounded by a layer of lymphocytes, mainly T cells, which help coordinate and sustain the immune response through cytokine signaling and macrophage activation. In more mature granulomas, an outer layer of fibroblasts and connective tissue may develop, helping to wall off the infected area and restrict further pathogen spread (36). A schematic representation of granuloma architecture is shown in **Figure 3**.

Granulomas are a hallmark of infection caused by the human pathogen *M. tuberculosis* (37, 38). Tuberculosis is an airborne infection and remains the leading cause of death from a single infectious agent worldwide (39). The harsh conditions within granulomas can drive *M. tuberculosis* into a slow-growing or dormant state associated with latent infection (40). These dormant bacteria exhibit reduced susceptibility to antibiotics, particularly those that target bacterial replication (40). Gaining deeper insight into host-pathogen interactions within granulomas, as well as the functional role of their architecture, is essential for the development of more effective strategies to achieve bacterial eradication.

Granulomas in tuberculosis have been studied using several experimental approaches. Tissue samples from patients allow analysis of the spatial distribution of cells and molecular components in well-developed granulomas (41, 42). However, such samples usually reflect later stages of disease, as they are often obtained during lung surgery or postmortem examination. Animal models, including non-human primates, rabbits, guinea pigs, and mice, make it possible to study granuloma formation and immune responses at earlier stages of infection and to manipulate specific pathways genetically or pharmacologically to better understand host-pathogen interactions (43). Furthermore, these *in vivo* models together with advanced imaging techniques, have been used to study the architecture and function of fully developed granulomas (44–46). However, they require tissue extraction and fixation to examine early cellular events and precursor structures. Additional drawbacks of animal models, including high cost and ethical concerns, have driven the development of more advanced *in vitro* models.

Although 2D *in vitro* culture systems have been used to study *M. tuberculosis* granulomas, they do not accurately reproduce the complex 3D environment encountered by the pathogen and host immune cells (47, 48). Therefore, 3D culture systems are more suitable for these studies. These 3D *in vitro* granulomas can be generated using scaffold-based approaches, such as hydrogels or mixtures of extracellular matrix components and synthetic polymers, or through the formation of spheroids and organoids (49, 50). In this context, a biomimetic 3D *in vitro* spheroid granuloma model was developed using patient-derived alveolar macrophages infected with *Mycobacterium bovis* bacille Calmette-Guérin (BCG), with magnetic nanospheres used to cluster the cells and form a macrophage spheroid (49). The addition of autologous adaptive immune cells from peripheral blood mononuclear cells resulted in their self-assembly around the spheroid, thereby generating a more organized and physiologically relevant granuloma-like structure (49). These spheroids showed a better control of bacterial growth than conventional monolayer cultures, emphasizing the importance of 3D architecture and multicellular interactions in host defense (49). An additional *in vitro* approach for investigating mycobacterial granulomas involves culturing human peripheral blood mononuclear cells with Sepharose beads coated with mycobacterial extracts, which serve as a scaffold to trigger granuloma-like organization (51). Another model uses collagen matrices containing fibroblasts, epithelial cells, and macrophages infected with *M. tuberculosis*, thereby better reflecting the complexity of the tissue microenvironment (52). Collagen-based microsphere constructs containing *M. tuberculosis*, human peripheral blood mononuclear cells and embedded macrophages have also been used to study antimicrobial resistance and the progression of immune responses over time (53). More advanced tissue-like models include collagen-supported 3D peripheral blood mononuclear cells systems that simulate *M. tuberculosis* dormancy, reactivation, and responses to anti-TNFα treatment during granuloma development (54). Collectively, these *in vitro* systems reproduce key biochemical and physiological features of *in vivo* granulomas, including cell-extracellular matrix interactions, multicellular organization, and gene expression patterns associated with mycobacterial infection.

However, several challenges still limit the ability of 3D *in vitro* models to fully replicate *in vivo* granulomas. A major limitation is the incomplete reproduction of biological complexity, as most models use only a limited number of cell types and fail to capture the dynamic recruitment and differentiation processes involved in granuloma development. This restricts their ability to represent the full spectrum of granuloma states, including latent and active disease. *In vivo* granulomas comprise gradients of oxygen, nutrients, and signaling molecules that strongly influence immune responses and pathogen behavior, but these are often poorly reproduced in *in vitro* 3D systems. Most models also lack vascular and lymphatic components, which are important for studying immune cell trafficking, systemic signaling, and drug delivery. In addition, biosafety constraints often require the use of surrogate strains instead of *M. tuberculosis*, which may reduce biological relevance.

### 2.3 Abscess

**Figure 4:**
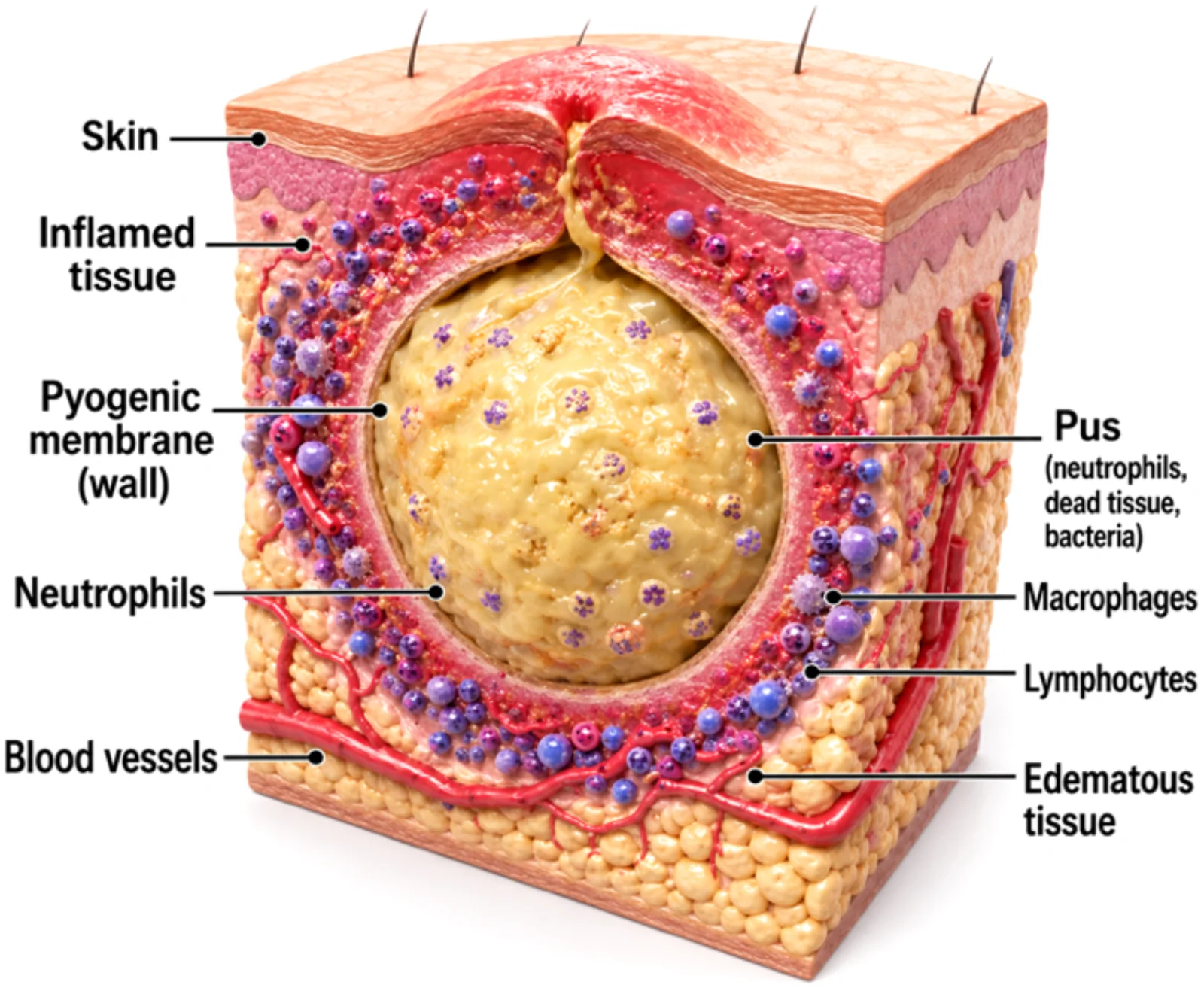
3D schematic representation of a superficial abscess. A central cavity filled with pus, composed of neutrophils, cellular debris, and bacteria, is surrounded by a membrane that forms the abscess wall. The adjacent tissue is inflamed and edematous, with infiltration by neutrophils, macrophages, and lymphocytes. Dilated blood vessels in the surrounding tissue reflect the local inflammatory response, while the overlying skin shows swelling and surface disruption above the lesion. This schematic representation was generated using AI-based tools.

An abscess is a localized accumulation of pus and necrotic tissue surrounded by inflammatory cells, such as macrophages and neutrophils, and enclosed by a fibrinous pseudocapsule. Abscesses may be superficial or may occur within deep tissues. Superficial abscesses develop near the skin surface, typically involving the epidermis and subcutaneous tissue (55). Deep organ abscesses form beneath the subcutaneous layer, including within systemic organs, and pose significant therapeutic challenges because of their concealed location and complexity (56, 57). A schematic representation of a superficial abscess is shown in **Figure 4**.

Abscess formation is a hallmark of many infections, including those caused by *S. aureus* (55, 58, 59). Although abscesses are host defense mechanisms that develop to confine pathogens, *S. aureus* can survive and replicate within these structures (60, 61), which may partly explain the frequent recurrence of infections observed in patients (62). Furthermore, the physicochemical conditions within abscesses, characterized by low pH, substantial debris accumulation, and reduced redox potential, can limit antibiotic diffusion and activity, contributing to recurrent or relapsing infection independently of classical resistance mechanisms (63, 64).

Abscesses are difficult to treat, highlighting the need for better antimicrobial strategies and improved disease models. Small animal models have been useful for investigating abscess formation in various tissues (55, 65). However, many pathogen virulence factors are human-specific, and therefore these models do not always reflect human disease accurately (66, 67). To overcome this limitation, 3D *in vitro* models incorporating human-derived cells and factors are increasingly being employed (64, 68). These models mimic abscess architecture, including gradients of oxygen, nutrients, and pH, making them valuable for investigating antimicrobial resistance, persistence, and drug penetration (64).

However, 3D models still have important limitations. One major limitation is the incomplete representation of the immune system. In most 3D models, only a limited number of immune cell types are included, and their behavior often differs from that *in vivo*. Key processes such as continuous immune cell recruitment, sustained inflammation, and pus formation are therefore not fully reproduced. In addition, abscess formation is a dynamic, multistage process that evolves over time, progressing from early infection to chronic inflammation or resolution (58). Most 3D *in vitro* models are limited to relatively short experimental periods and cannot capture long-term progression or recurrence.

## 3 Advanced *in vitro* approaches for studying infection in 3D

Animal models and conventional 2D cell cultures have significantly advanced our understanding of host-pathogen interactions, but they often fail to reflect key aspects of human infections (69, 70). For instance, 2D epithelial monolayers lack a proper integration within an extracellular matrix network and therefore do not accurately reflect the architecture or polarity of intact epithelial tissues found *in vivo* (10). The 3D cell culture systems can overcome some of these limitations by supporting cell-cell and cell-extracellular matrix interactions that help maintain tissue specificity and homeostasis (71–73). These models create a more physiologically relevant setting for investigating host-pathogen dynamics (69, 70). They also support translational research by enabling the testing of new drugs and supporting personalized therapeutic approaches.

**Figure 5:**
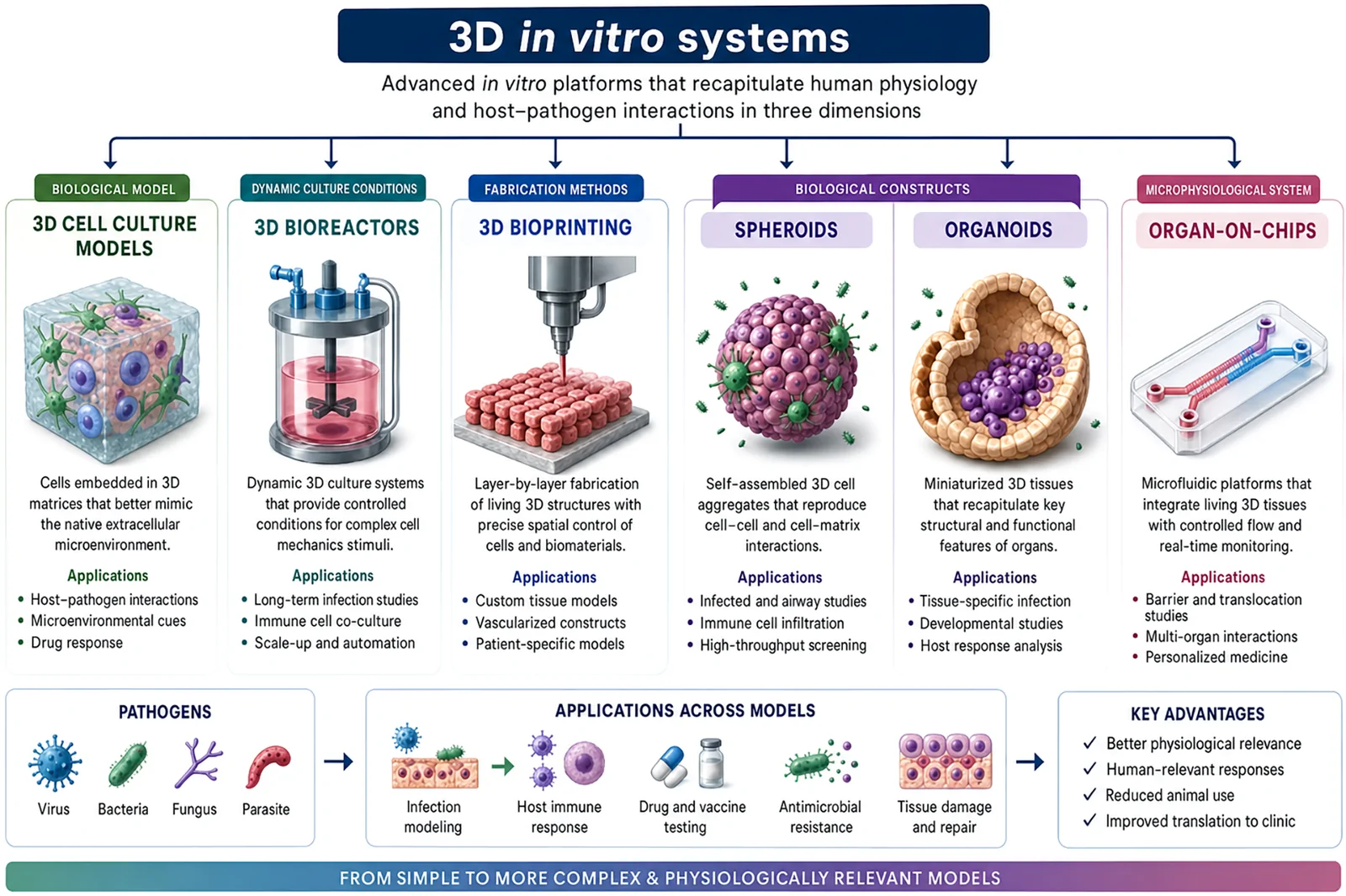
Conceptual illustration of 3D *in vitro* systems. 3D *in vitro* platforms include 3D cell culture models, in which cells are embedded in extracellular matrix-like scaffolds; bioreactors, which provide dynamic culture conditions such as controlled perfusion and mechanical stimulation; and 3D bioprinting, which enables the fabrication of spatially defined multicellular structures. In addition, spheroids are self-assembled cell aggregates that mimic cell-cell and cell-matrix interactions, whereas organoids are structures that recapitulate key architectural and functional features of native tissues. Organ-on-chip systems integrate microfluidics with living tissues to model physiological flow and inter-organ communication. This conceptual illustration was generated using AI-based tools.

3D *in vitro* systems encompass a range of approaches, including 3D cell culture models and bioreactors, 3D bioprinting, spheroids, organoids, and miniaturized models known as organ-on-chip systems. Conceptually, spheroids and organoids are biological constructs, 3D bioprinting is a fabrication method, bioreactors provide dynamic culture conditions, and organ-on-chip models are microfluidic platforms. Across these models, pathogens including viruses, bacteria, fungi, and parasites, can be used to investigate infection mechanisms, host immune responses, drug and vaccine efficacy, antimicrobial resistance, and tissue damage and repair. A conceptual illustration of these systems is shown in **Figure 5**. The following sections provide a detailed overview of these systems.

### 3.1 3D cell culture models and bioreactors

Cells can be cultured in 3D using scaffold-based systems made from synthetic or natural material, as well as hydrogels (74). These materials mimic the extracellular matrix, which provides signals that regulate cell behavior, growth, and differentiation (75). Collagen is commonly used to support cell attachment within matrices (75). Hydrogels are another key component of 3D systems, and variations in their composition have been explored to better understand how they influence cell spreading, proliferation, and mesenchymal stem cell differentiation (75).

**Figure 6:**
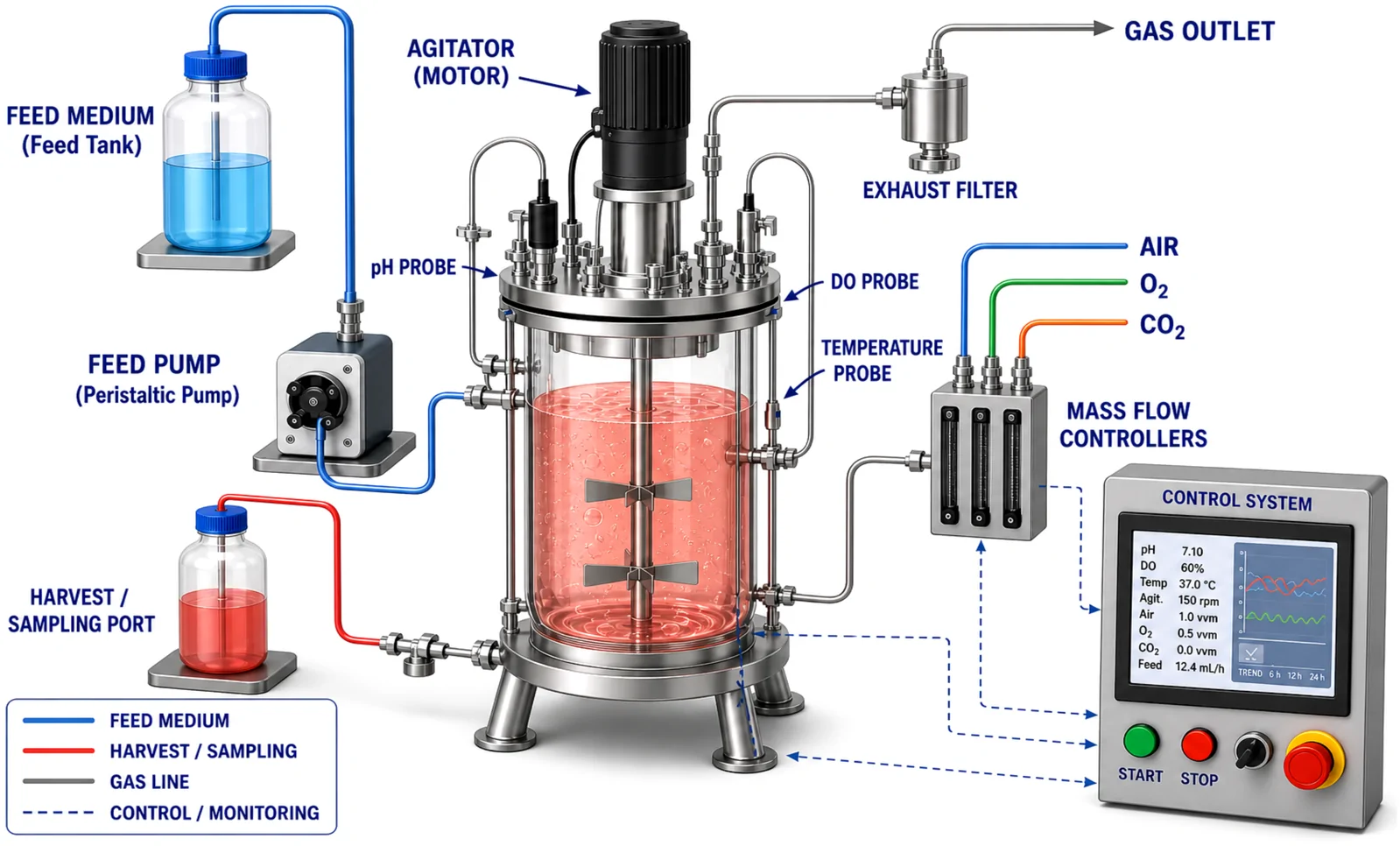
Schematic representation of a bioreactor system for 3D cell culture. The diagram illustrates a stirred-tank bioreactor operated in fed-batch mode. Fresh medium is supplied from a reservoir via a peristaltic pump, enabling controlled nutrient addition during cultivation. The bioreactor vessel is equipped with an impeller driven by an overhead motor to provide mixing and mass transfer. Key process parameters are monitored using integrated probes, including pH, dissolved oxygen (DO), and temperature sensors. A sampling/harvest port allows periodic withdrawal of culture for analysis or product recovery. System operation, monitoring, and control are managed via an external control unit, which displays real-time process parameters and regulates feeding, aeration, and agitation. This schematic representation was generated using AI-based tools.

A bioreactor is a system designed to support 3D cell culture under dynamic conditions, enabling the application of mechanical, physical, and chemical stimuli to biological samples and the assessment of their effects (76, 77). The capacity to apply dynamic flow and facilitate nutrient delivery enables these systems to recreate a microenvironment similar to that found *in vivo* (78, 79). There are several types of bioreactors, including spinner flasks and rotating wall vessels. In spinner flasks, cells are kept in suspension by continuous stirring that maintains constant circulation of the culture medium (79–81). Scaffolds are positioned within the flask, and the continuous movement of the medium facilitates cell attachment to the support, helping to develop a tissue-like architecture (79). These systems are often run in batch mode, which reduces contamination risk but limits culture duration due to the accumulation of metabolites and waste over time (79). Continuous culture and fed-batch systems allow medium replenishment and partial removal of cellular waste, although the risk of external contamination remains (79). A schematic representation of a bioreactor system is shown in **Figure 6**.

Rotating wall vessels consist of a horizontally rotating cylindrical chamber equipped with an oxygenation system that ensures adequate gas exchange (82). The vessel is filled with culture medium, and as it rotates, the cells remain in suspension, thereby minimizing shear forces and sedimentation while supporting three-dimensional cell growth (82, 83).

However, 3D cell culture models and bioreactors face significant biological, technical, and practical limitations. They require precise control of environmental parameters such as pH, oxygen levels, and flow rates. Even small variations in these conditions can lead to substantial differences in experimental outcomes, contributing to reproducibility issues across experiments and laboratories. In addition, scaling these systems for industrial or clinical applications is challenging, as it is difficult to maintain uniform conditions throughout larger volumes. Furthermore, they often require specialized materials, equipment, and expertise, as well as longer preparation and culture times.

### 3.2 3D bioprinting

**Figure 7:**
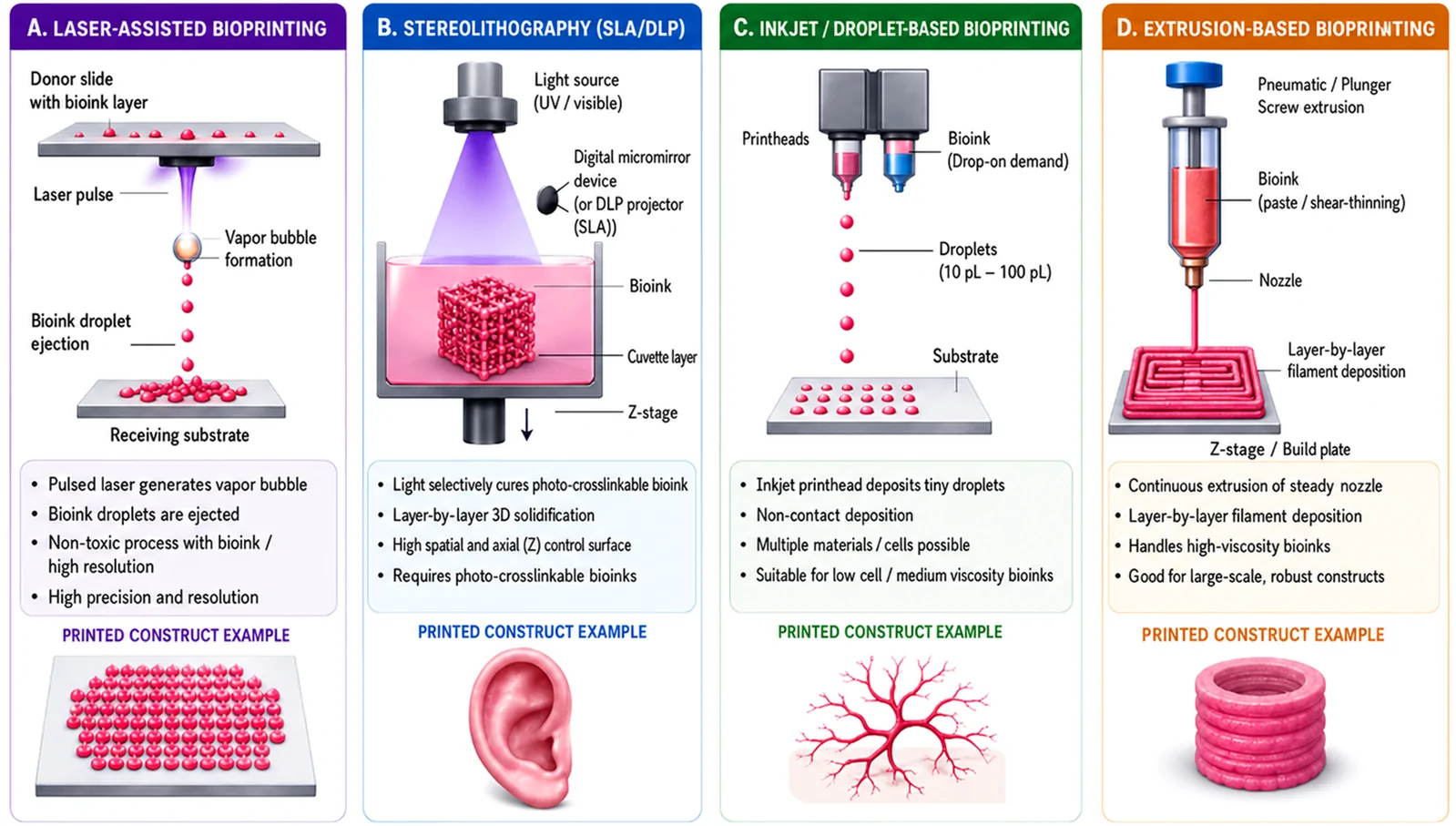
Conceptual illustration of the main 3D bioprinting methods. **(A)** Laser-assisted bioprinting uses laser pulses to transfer droplets of bioink onto a substrate with high resolution while maintaining cell viability. **(B)** Stereolithography employs light-based photopolymerization to solidify photosensitive bioinks layer by layer into defined 3D structures. **(C)** Inkjet (droplet-based) bioprinting deposits controlled droplets of low-viscosity bioinks in a defined pattern, enabling rapid and cost-effective fabrication. **(D)** Extrusion-based bioprinting uses mechanical or pneumatic forces to continuously dispense bioink filaments through a nozzle, allowing the construction of larger cell-laden structures. This conceptual illustration was generated using AI-based tools.

3D bioprinting uses layer-by-layer printing of living cells and supportive materials to create biological structures such as tissues and, potentially, whole organs (84, 85). It enables the production of complex 3D tissue models for studying disease mechanisms, testing new drugs, and developing personalized therapeutic approaches, and for investigating host-pathogen interactions (86, 87). A key component of this technology is bioink, the material from which bioprinters create living tissues (88). Bioinks typically consist of hydrogels, living cells, and biomolecules that provide structural support for cell growth and interactions (88). Several bioprinting modalities have been developed (89), including laser-assisted bioprinting, which uses laser pulses to transfer small droplets of bioink onto a substrate (90); stereolithography, which uses light, usually ultraviolet, to solidify bioinks layer by layer into precise 3D biological structures (91); inkjet (or droplet-based) bioprinting, which deposits small droplets of bioink containing cells and biomaterials onto a substrate in a controlled pattern (92); and extrusion-based bioprinting, which uses mechanical or pneumatic forces to dispense bioink through a nozzle that follows a computer-generated pattern (93). A conceptual representation of these systems is shown in **Figure 7**.

**Figure 8:**
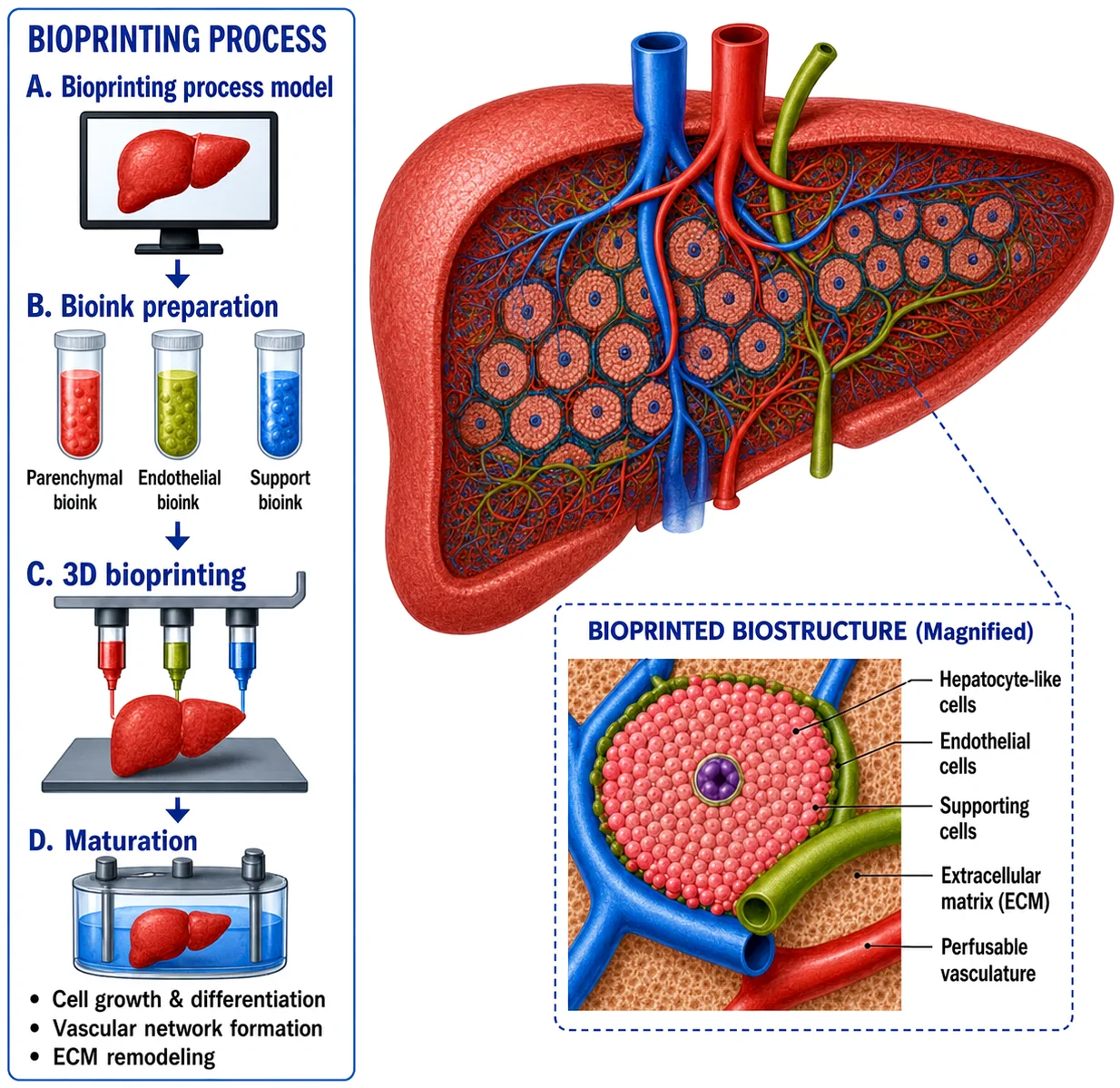
Schematic representation of the bioprinting process for engineering liver tissue. Left panel: sequential steps including **(A)** generation of a digital liver model, **(B)** preparation of multiple bioinks containing parenchymal, endothelial, and supporting cells, **(C)** layer-by-layer 3D bioprinting, and **(D)** post-printing maturation involving cell growth, differentiation, vascular network formation, and extracellular matrix remodeling. Right panel: representation of the bioprinted liver construct with integrated vascular-like structures. Inset: magnified view of the bioprinted microstructure showing hepatocyte-like cells, endothelial cells, supporting cells, extracellular matrix components, and perfusable microvessels. This schematic representation was generated using AI-based tools.

3D bioprinting has been used to engineer diverse tissue models, including liver, bone, vascular, cardiac, and lung constructs (94). A schematic representation of a liver model generated by 3D bioprinting is shown in **Figure 8**.

Bioprinted tissue models are increasingly being used to study viral and bacterial infections (95, 96). For example, 3D bioprinting has been employed to generate mature bacterial biofilms from alginate-based bioinks containing clinically relevant species, including *Escherichia coli*, *S. aureus*, and *P. aeruginosa*, and these models have been used for antimicrobial testing (97). Furthermore, several 3D-printed models have been developed to investigate viral infections, particularly in the context of the COVID-19 pandemic and influenza (96, 98). These include systems designed to mimic epithelial barriers, ranging from general epithelial infection models to more specialized representations of the alveolar, blood-retina, and nasal barriers, as well as more complex tissue models of the lung, liver, and neural tissues (99–103). However, several limitations remain, including the incomplete replication of systemic immune responses, vascular perfusion, long-term disease progression, and whole-organ physiology. In addition, suitable bioinks that meet the required mechanical and biological criteria are still limited, making the development of new bioink formulations a key focus in the field.

### 3.3 Spheroids

Spheroids are 3D cell clusters formed by the self-organization of suspended cells under non-adherent conditions (104–106). They can consist of one or multiple cell types while preserving cell-cell interactions and tissue-specific features (104–107). Their densely packed structure also creates natural gradients of nutrients, oxygen, and pH, making them more representative of *in vivo* conditions (108, 109). Spheroid formation occurs through aggregation, compaction, and growth, driven by cell-cell and cell-matrix interactions, particularly via integrins (110). As they grow, spheroids develop increasingly complex cellular architectures that more closely reflect the *in vivo* microenvironment.

**Figure 9:**
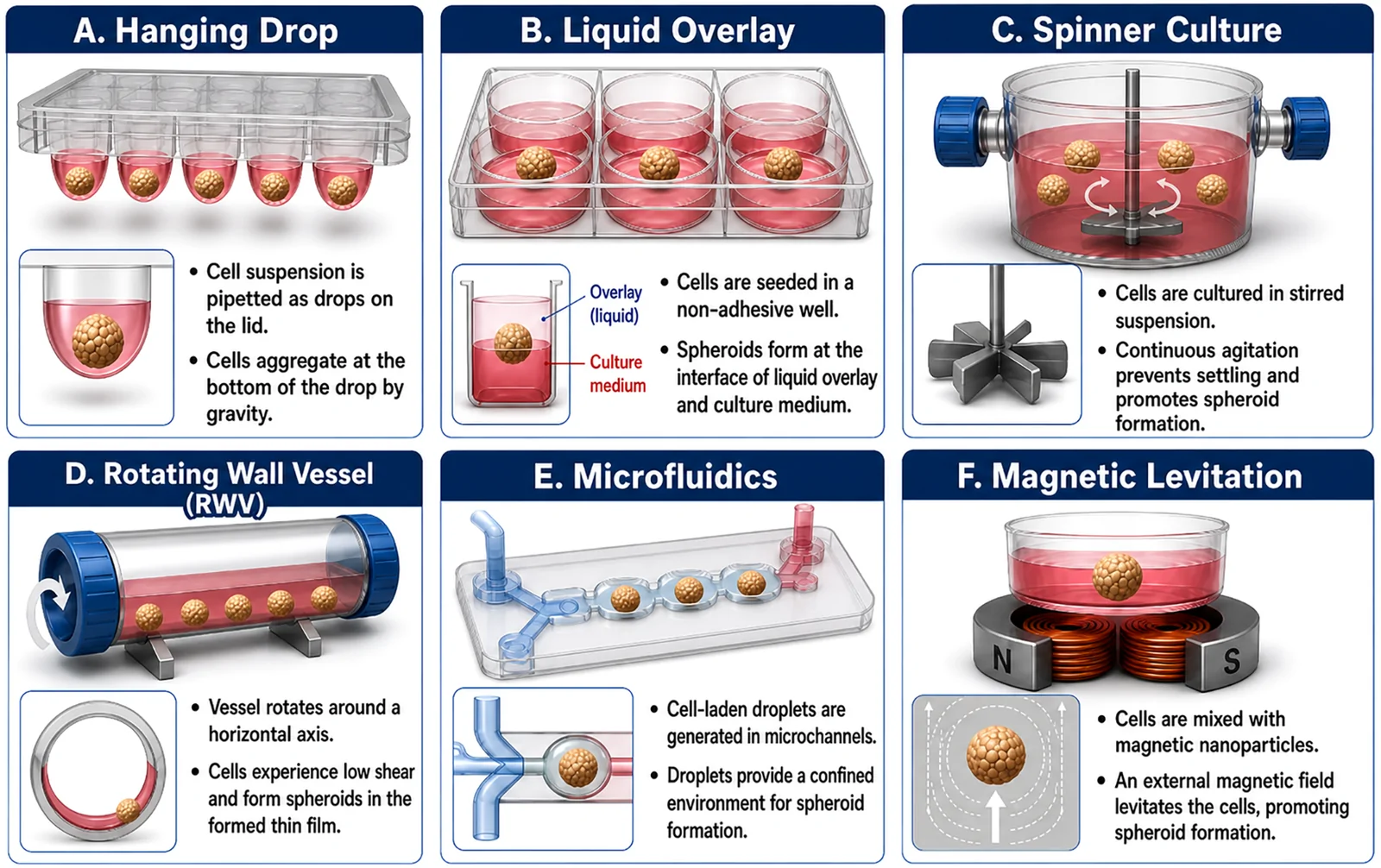
Conceptual illustration of the different methods for generating spheroids. **(A)** Hanging-drop method: Cell suspensions are pipetted as droplets onto the lid of a culture plate. Upon inversion, gravity drives cell aggregation at the bottom of each droplet, thereby promoting spheroid formation. **(B)** Liquid overlay: Cells are seeded in non-adhesive wells, where spheroids form in suspension at the liquid interface. **(C)** Spinner culture: Cells are cultured in stirred suspension; continuous agitation prevents settling and promotes spheroid formation. **(D)** Rotating wall vessel: The vessel rotates around a horizontal axis, creating a low-shear environment that supports spheroid formation within the culture medium. **(E)** Microfluidics: Cell-laden droplets are generated in microchannels, providing a confined environment for spheroid formation. **(F)** Magnetic levitation: Cells are mixed with magnetic nanoparticles and levitated by an external magnetic field, thereby promoting spheroid formation. This conceptual illustration was generated using AI-based tools.

Spheroids can be generated using several techniques, including the hanging-drop method, liquid overlay systems, and microfluidic technologies (106). A conceptual representation of these approaches is shown in **Figure 9**. The choice of approach depends on the specific application and the desired properties of the resulting spheroids.

In the hanging-drop technique, cells aggregate within suspended small droplets, producing highly uniform spheroids without specialized equipment, although evaporation can be a limitation (111, 112). In the liquid overlay method, non-adhesive coatings prevent cell attachment and promote spheroid formation, offering a simple low-cost approach but often with greater size variability (104, 113). Dynamic systems, such as spinner flasks, keep cells in suspension through continuous agitation, enabling large-scale spheroid production but often causing shear stress and heterogeneity (104, 114). A lower shear stress alternative is the rotating wall vessel system, which maintains cells in suspension by continuous rotation (115–117). The rotating wall vessel bioreactor provides adequate oxygenation and nutrients to support cellular development and polarization (116, 118). Microfluidic technologies enable robust, reproducible, and high-throughput formation of 3D cell spheroids that have been used in drug screening, disease modeling, and tissue engineering (119, 120). Microfluidic approaches include microwell-based systems, which generate uniform spheroids in controlled microenvironments, and droplet-based systems, which encapsulate cells in droplets for high-throughput spheroid production (121–123).

Magnetic levitation offers another strategy, using magnetic nanoparticles to promote cell aggregation and spheroid formation, leading to compact 3D structures with features similar to native tissues (124, 125).

Spheroids can model a variety of tissues and organs, including tumors (126), neural structures (127, 128), and liver tissue (129), making them highly valuable for studying tissue-specific functions and underlying disease processes. In this context, spheroid-bacteria co-culture models have been developed to evaluate the permeability and efficacy of antimicrobial agents (130). In addition, immune-infiltrated cancer spheroid models have been established by continuously perfusing immune cells into a cancer spheroid using a gravity-driven flow, thereby enabling the study of immune cell infiltration and differentiation in the tumor microenvironment (131). However, a major limitation of spheroids is that their formation is influenced by numerous factors, including the cell type used, culture conditions, medium volume and composition, and cell seeding density, which can reduce reproducibility (132). Additional limitations include low cellular complexity, lack of vasculature and circulation, artificial oxygen and nutrient gradients, and limited tissue architecture and polarity, all of which can affect how well they replicate *in vivo* conditions.

### 3.4 Organoids

Organoids are 3D cell structures grown *in vitro* from stem cells or tissue-specific progenitor cells that can self-organize to resemble the structure and function of native organs. Compared with conventional cell culture models, they show greater cellular and structural complexity while remaining suitable for long-term experiments, advanced analysis, and higher-throughput applications (133, 134). The concept of generating organoids was first demonstrated by showing that embryonic stem cells can self-organize into cortical neuroepithelial structures with organized architecture and polarity in culture (135). Later, adult stem cells were shown to expand into complex 3D structures resembling native organs (136), followed by successful derivation of similar structures from human induced pluripotent stem cells (137).

Organoids can be generated either directly from tissue, commonly referred to as tissue stem cell (TSC)-derived organoids or from pluripotent stem cells (PSCs), including embryonic and induced PSCs (137–139). TSC-derived organoids usually arise from tissue-resident stem or progenitor cells of a single germ layer, most often the endoderm, whereas PSC-derived organoids can form more complex multilineage structures containing diverse cell types (139). Recent technological advances have further improved organoid models through multicellular co-culture systems, organ-specific extracellular matrices, genome editing with CRISPR-Cas9, and the introduction of vascular-like networks, increasing their value for disease modeling and therapeutic testing (140–143).

Organoids have demonstrated substantial value in developmental biology, disease modeling, oncology, drug discovery, and the study of host-pathogen interactions (144–150). They are especially useful for studying human-specific pathogens, especially when suitable animal models are unavailable or when replication depends on specialized host cell types. Several examples of organoid-based models are outlined below.

#### 3.4.1 Intestinal organoids

**Figure 10:**
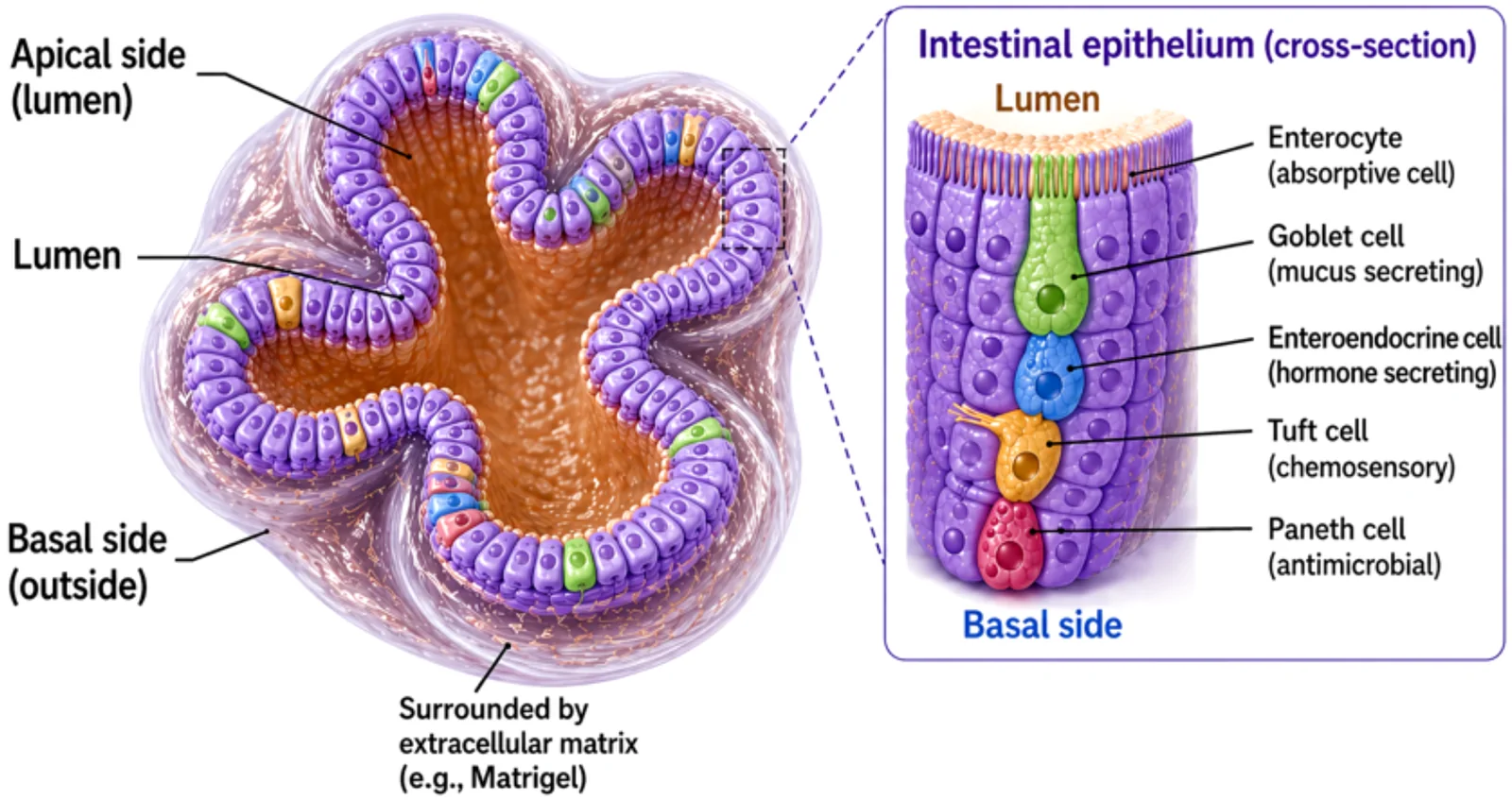
Schematic representation of an intestinal organoid. The left panel shows a 3D intestinal organoid with a polarized epithelium surrounding a central lumen. Crypt-like buds extend outward, resembling the organization of the intestine *in vivo*. The right panel shows a cross-section of the intestinal epithelium highlighting major differentiated cell types. This schematic representation was generated using AI-based tools.

Intestinal organoids closely replicate the architecture and function of the human intestine (151, 152). Advances in preserving intestinal stem cell identity have enabled the establishment of 3D culture systems that form complex multicellular structures with crypt-villus organization and physiologically arranged cell types such as Paneth and enteroendocrine cells (153). These models reproduce key intestinal functions, including mucus secretion, enzymatic activity, oxygen gradients, and spatially regulated antimicrobial peptides (153). A schematic representation of an intestinal organoid is shown in **Figure 10**.

In infection research, intestinal organoids are valuable for studying host-bacteria interactions, such as Salmonella (154–156), host-virus interactions (157), and the gastrointestinal microbiota (158). Importantly, human intestinal organoids can also be cryopreserved at different stages of expansion, enabling the establishment of biobanks (159).

#### 3.4.2 Lung organoids

**Figure 11:**
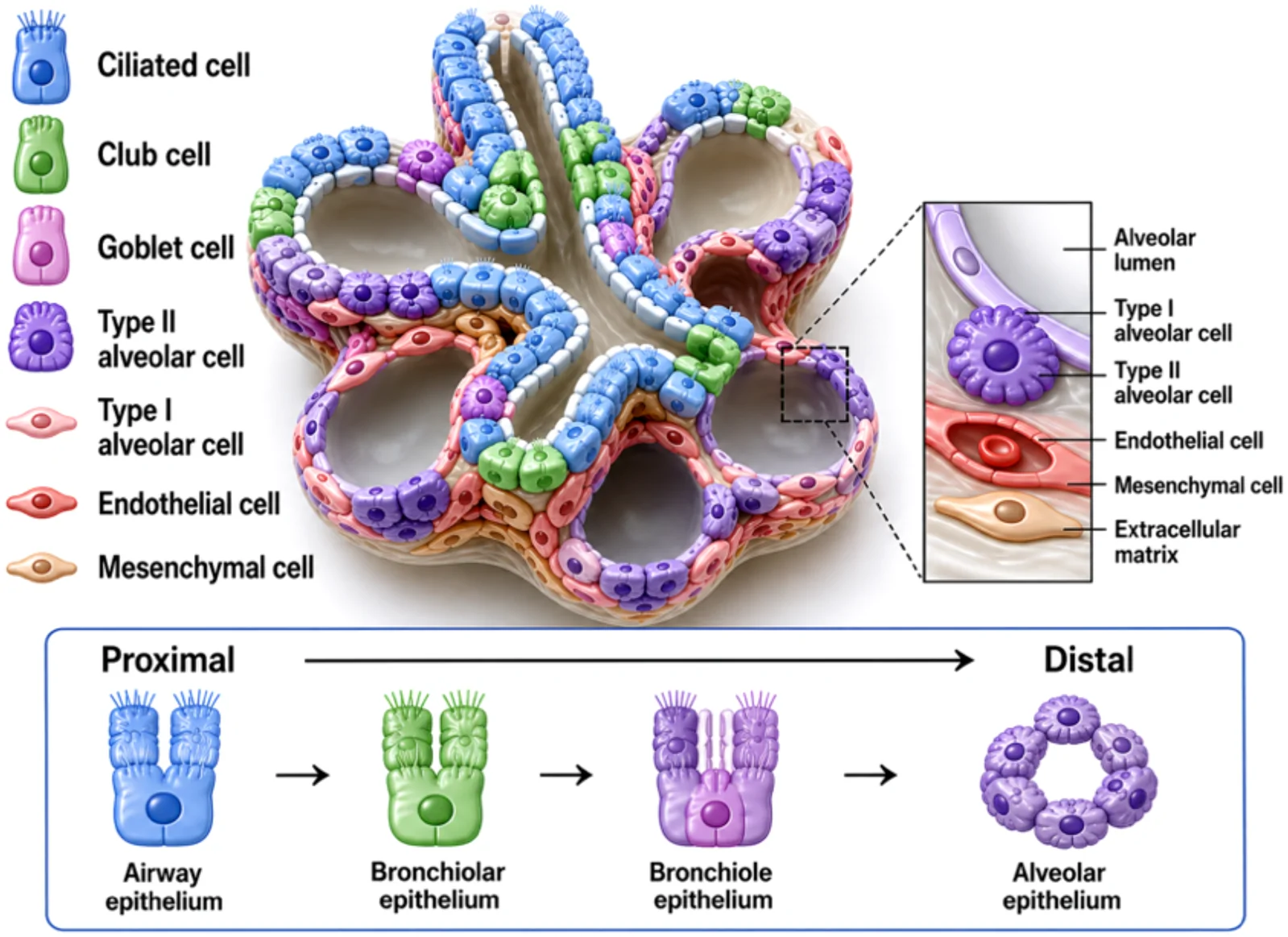
Schematic representation of a lung organoid. The upper panel shows a representative lung organoid composed of multiple epithelial lineages organized around a branched lumen. Distinct cell types are illustrated and color-coded. An inset highlights epithelial ultrastructure and spatial relationships. The lower panel illustrates a developmental trajectory along the proximal-distal axis. Proximal airway-like regions contain airway and bronchiolar epithelium enriched in secretory and ciliated cells, transitioning distally toward bronchioles and alveolar structures composed predominantly of type I and type II alveolar cells. This schematic representation was generated using AI-based tools.

Lung organoids are important models for respiratory disease research (160, 161). They can be generated from adult tissue after enzymatic digestion (162), embryonic progenitor cells (163), or induced pluripotent stem cells (163, 164). Although lung organoids do not yet fully reproduce the cellular diversity of native lung tissue, ongoing efforts to incorporate additional cell types, such as macrophages, alveolar type II cells, and endothelial cells, continue to improve their physiological relevance (165–167). A schematic representation of a lung organoid is shown in **Figure 11**.

Lung organoids are valuable for studying viral and bacterial infections. They were among the first models used to investigate SARS-CoV-2 pathogenesis, including a distal lung organoid with apical-out polarity that exposes ACE2 and enable efficient infection of AT2 cells (168–171). Lung organoids have also been used to study influenza virus infection mechanisms and screen antiviral drugs (172–174). With respect to bacterial infections, only a limited number of studies have used lung organoid systems to model bacterial pathogens, particularly mycobacteria. They offer a promising platform to examine interactions between *M. tuberculosis*, airway epithelium, and macrophages in a physiologically relevant setting (175–177).

#### 3.4.3 Other organoids

Kidney organoids are primarily generated from pluripotent stem cells, including embryonic stem cells and induced pluripotent stem cells, which can self-organize into structures resembling nephrons and recapitulate key features of early kidney development (143, 178).

Cardiac organoids are self-organizing 3D systems composed of progenitor cells, cardiomyocytes, endothelial cells, and fibroblasts that closely resemble key features of the heart *in vivo* (179–181). They have been used to model congenital heart defects and have provided valuable insights into heart development, cardiovascular disease, and drug-induced cardiotoxicity (179–181).

Cerebral organoids are generated from embryonic stem cells or induced pluripotent stem cells and provide versatile models of human brain development (182–184). Depending on the method, they can be produced either by unguided differentiation, yielding mixed regional identities, or by guided differentiation with morphogens, resulting in more uniform and reproducible structures (182, 183). These different strategies enable the generation of organoids tailored to specific experimental needs. Combining patterned organoids into assembloids further enables the study of interactions between brain regions (185), interneuron migration from ventral to dorsal areas (186), and the formation of axonal connections (187). Cerebral organoids are also valuable tools for studying neurodegenerative disorders such as Alzheimer’s disease (188), and viral infections such as Zika virus (189), including its effects on cortical progenitors and microcephaly-like phenotypes (190, 191).

A major limitation of many organoid models is their resemblance to fetal or immature tissue rather than fully differentiated adult organs. This immaturity can affect susceptibility to infection and reduce their accuracy for modeling disease in adults. To address this, strategies such as mechanical or biochemical stimulation are being explored to promote maturation. Current research also aims to improve physiological relevance by incorporating immune cells, endothelial cells for vascularization, and commensal microbiota into organoid systems. In parallel, protocol standardization and automation are being developed to enhance reproducibility and scalability, especially for drug screening.

#### 3.4.4 Generation of immune-competent organoids

As already mentioned, a major limitation of conventional organoid models is the lack of a tissue-specific immune compartment. Incorporating immune cells is therefore crucial for generating more physiologically relevant organoids. Progress in this area includes organoids co-cultured with blood-derived innate and adaptive immune cells (192, 193). One example is the generation of human intestinal immune-organoids formed by self-assembly of epithelial cells with autologous tissue-resident memory T cells, a subset of which integrates into the epithelium and supports barrier surveillance (194).

Four main strategies are currently used to incorporate immune cells into organoid models. First, immune and stromal cells can be embedded together within a scaffold, allowing defined cell populations of immune cells to be included during organoid formation, and enabling controlled reconstruction of the microenvironment with more physiologically relevant cell-cell interactions (195). Second, isolated immune cells can be co-cultured with pre-established organoids to study their effect on organoid development and function (196). For example, co-culturing tumor organoids with immune cells provides a means to study how immune cells influence tumor growth and progression (197). Third, immune cells can be introduced directly into the organoid lumen to more closely mimic physiological exposure at epithelial surfaces (198). Fourth, explant tissues can be cultured while preserving their native resident immune cells, providing a model that maintains important features of the original tissue microenvironment and cellular composition (199).

Incorporating immune components is critical to make organoids more physiologically relevant and improves the study of infection, inflammation, host-pathogen interactions, and immunomodulatory therapies, vaccines, and personalized treatments.

### 3.5 Organ-on-chip devices

Organ-on-chip devices are miniaturized engineered systems that mimic the structure and function of human organs (200). By combining microfluidic technology with tissue engineering, they reproduce key physiological organ functions and support applications such as drug screening and real-time administration of drugs or microorganisms (201–203). The integration of flexible polymer membranes and mechanical stimuli, such as breathing- or peristalsis-like motions, enables organ-on-chip systems to model organ function and disease more accurately (204, 205). Active perfusion further allows immune cells to be introduced into endothelial-lined channels, enabling circulation in a manner analogous to blood flow (206, 207). This design more closely mimics vascular perfusion and transport of drugs and metabolites across the endothelium (208). Organ-on-chip systems can also generate oxygen gradients that support co-culture of complex microbiomes, including both anaerobic and aerobic bacteria, on human epithelial layers for several days (209).

**Figure 12:**
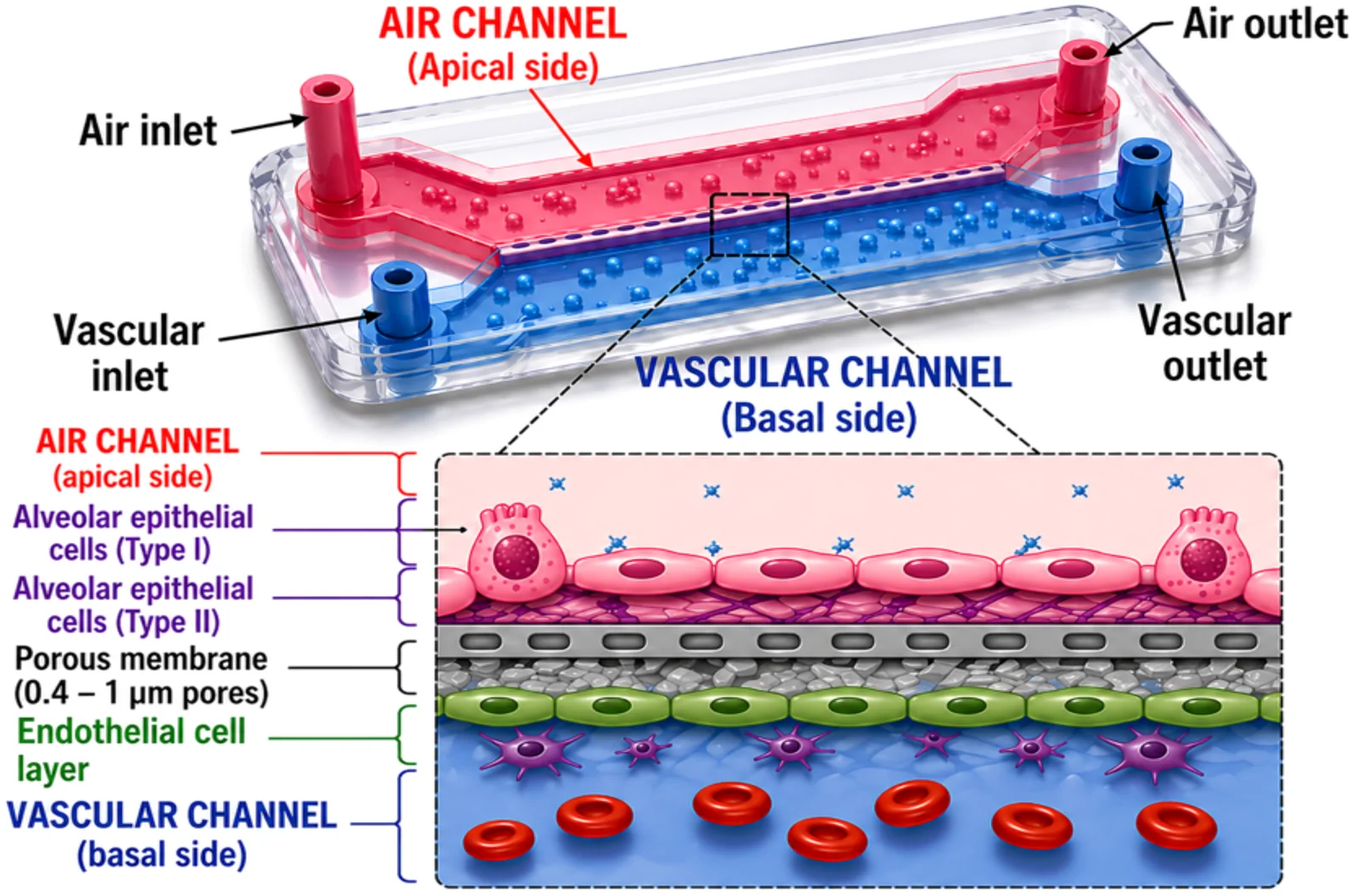
Schematic representation of a lung-on-chip. The system consists of two parallel microchannels separated by a porous membrane. The air channel (apical side) is lined with alveolar epithelial cells and exposed to air, enabling the formation of an air-liquid interface and surfactant production. The vascular channel (basal side) is lined with endothelial cells and perfused with culture medium containing circulating components such as red blood cells, mimicking blood flow. The porous membrane allows for gas exchange, molecular transport, and cell-cell signaling between the epithelial and endothelial layers while maintaining compartmentalization. Inset: Magnified cross-sectional view illustrating the layered architecture, including the alveolar epithelium, porous membrane, endothelial layer, and perfused vascular channel. This schematic representation was generated using AI-based tools.

Organ-on-chip systems that reconstitute organs such as lungs (210, 211), liver (212), intestine (213), kidneys (214), brain (215), as well as interconnected multi-organ systems (216), have been developed to investigate human disease mechanisms. For example, a lung alveolus-on-chip model composed of primary human alveolar epithelial and pulmonary microvascular endothelial cells has been used to study pulmonary thrombosis by perfusing whole blood through the vascular compartment (217). A lung airway-on-chip coated with primary cystic fibrosis bronchial epithelial cells has also been used to study the recruitment of immune cells in response to *P. aeruginosa* (218). Furthermore, lung-on-chip devices have been widely applied to investigate respiratory viral infections (219–221), including SARS-CoV-2 (222). A schematic representation of a lung-on-chip is shown in **Figure 12**.

Intestine-on-chip systems with highly differentiated human villus structures have been developed using continuous fluid flow and peristalsis-like motions (223). In these models, co-culture of human intestinal epithelial cells with aerobic and anaerobic gut microbiota under controlled oxygen gradients showed that a transluminal hypoxia gradient improves barrier function and maintains microbial diversity (209).

A microfluidic glomerulus-on-a-chip has been generated that reproduces key features of the native glomerular unit, and has been used to study hypertensive kidney disease (214). In addition, a distal tubule kidney-on-chip model has been used to investigate renal dysfunction caused by Pseudorabies virus infection (224).

For the liver, a microfluidic liver-on-chip based on human primary hepatocytes and non-parenchymal cell types has been applied to study hepatitis B virus infection (225). This model successfully recapitulates key features of the hepatic sinusoid, including bile canaliculi formation and proper cellular polarization (225).

Brain-on-chip systems have likewise been generated to model neurological diseases. For example, a device incorporating dopaminergic neurons, astrocytes, microglia, pericytes, and brain microvascular endothelial cells cultured under fluid flow has been used to investigate endothelial dysfunction in Parkinson’s disease, reproducing features such as phosphorylated α-synuclein accumulation, mitochondrial dysfunction, neuroinflammation, and barrier impairment (226). Numerous blood-brain barrier chip models have also been established to mimic *in vivo*-like permeability and support studies of neuroinflammation, transport across the blood-brain barrier, and pathogen invasion of the brain, including *Cryptococcus neoformans* tropism and barrier crossing (227, 228).

**Figure 13:**
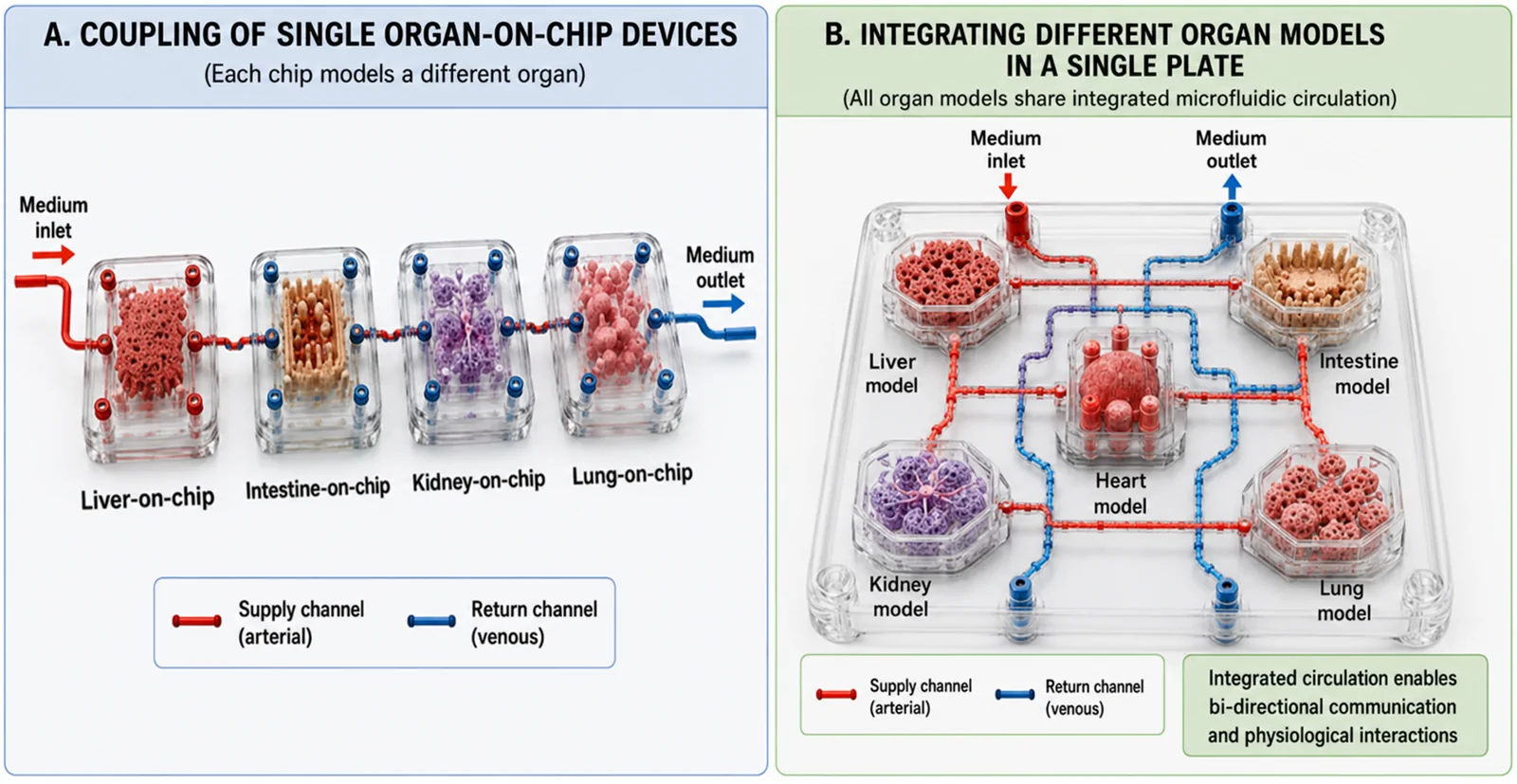
Conceptual illustration of strategies for generating multi-organ-on-chip systems. Two main approaches are illustrated for constructing multi-organ platforms. **(A)** Coupling of individual organ-on-chip devices: Separate chips, each representing a specific organ, are fluidically interconnected in series via microchannels, allowing sequential medium flow from inlet to outlet and enabling inter-organ communication through circulating factors. **(B)** Integration of multiple organ models within a single plate: Distinct organ compartments are incorporated into a unified microfluidic device that shares a common circulation network, often including a central pump (e.g., a heart model), enabling recirculating flow and bidirectional interactions between tissues. Red channels indicate supply (arterial-like) flow, and blue channels indicate return (venous-like) flow. This conceptual illustration was generated using AI-based tools.

More recently, organ-on-chip technology has advanced toward integrated multi-organ systems that combine the functions of several tissues within one platform (216, 229–234). These multi-organ-on-chip models are created either by fluidically connecting individual organ-on-chip units or by integrating several organ models within a single plate (233). Both strategies enable investigation of organ-organ interactions and drug responses at the whole-body level. A conceptual illustration of these approaches is presented in **Figure 13**.

Multi-organ-on-chip systems are valuable for toxicity screening (235, 236), assessing prodrugs that become active only after undergoing hepatic metabolism (237), and pharmacokinetic studies designed to understand and predict biological responses (238, 239), among other applications. However, major challenges remain in maintaining the homeostasis of multiple organ systems and in incorporating all essential physiological factors, such as hormonal signaling, lymphatic function, the microbiome, and proper innervation.

### 3.6 Innovative analytical tools

Traditional bulk approaches, which average cellular activity across large cell populations, may obscure critical host-pathogen interactions and immune responses occurring within defined tissue microenvironments. Recent advances in spatial transcriptomics and spatial proteomics make it possible to study infection patterns across tissues, cell types, and disease stages with much greater precision (240). Spatial transcriptomics enables the measurement of gene expression while preserving the spatial organization of cells in tissues (241). Two major spatial transcriptomic approaches have been developed. Imaging-based approaches detect RNA directly in intact tissue sections using microscopy and fluorescent probes, as in single-molecule fluorescence *in situ* hybridization, seqFISH, and MERFISH (242–244). Their main advantage is very high spatial resolution, often at single-cell or subcellular level, although they are typically limited to selected genes and require intensive imaging and analysis. In contrast, sequencing-based approaches capture RNA on spatially barcoded arrays or beads and identify transcripts through next-generation sequencing, as in platforms such as 10x Visium and Slide-seq (245, 246). These methods provide broader and often near-whole-transcriptome coverage, but usually at lower spatial resolution, meaning that one capture spot may contain RNA from multiple cells and require computational deconvolution (245, 246). Together, these approaches are complementary: imaging-based methods provide high-resolution *in situ* mapping, whereas sequencing-based methods are better suited for large-scale exploratory studies. A major innovation in this field is the simultaneous analysis of host and pathogen transcriptional signatures, which enables an integrated spatial view of infection, improves predictive modeling of disease progression, and helps identify previously unrecognized virulence determinants and mechanisms of pathogen adaptation (247).

Spatial proteomics extends these insights by mapping the distribution and abundance of proteins, which more directly reflect functional cellular states than RNA alone (248, 249). Built on advances in mass spectrometry, imaging, and computational analysis, spatial proteomics can reveal signaling activity, receptor expression, post-transcriptional regulation, and cell–cell communication *in situ* (248, 249). In infection research, these approaches help define how cytokines, checkpoint molecules, antimicrobial effectors, and tissue-remodeling factors are organized across lesions or inflamed sites, providing a more direct view of functional heterogeneity and the biological mechanisms underlying infection (248, 249).

## 4 Current limitations and future directions of 3D *in vitro* systems

Although 3D *in vitro* systems have advanced considerably in recent years, they still face important limitations that constrain their physiological relevance and translational value. The incorporation of immune cells and vascularization remains incomplete, limiting the ability of many models to reproduce innate immune cell recruitment and multicellular crosstalk during infection. Modeling adaptive immunity is even more challenging, although some advanced co-culture systems have incorporated T cells or peripheral blood mononuclear cells (197, 250, 251). Adaptive immune responses are difficult to sustain and standardize *in vitro* because they depend on complex cell communication, antigen-specific activation, and prolonged culture periods. In addition, donor-to-donor variability in primary or stem cell-derived systems introduces substantial heterogeneity. Antigen presentation can be partially modeled by including dendritic cells, macrophages, or other antigen-presenting cells, but fully reproducing robust and durable antigen-specific adaptive immune responses remains difficult (251, 252).

Reproducibility across laboratories is another persistent concern due to variations in scaffold materials, culture conditions, fabrication methods, and analytical endpoints. Biosafety constraints also limit the use of complex 3D infection models with high-risk pathogens, particularly when long-term co-culture or advanced live imaging is required. In addition, validation remains difficult because benchmarking these systems against human infection pathology is often constrained by limited patient tissue, incomplete clinical correlates, and the complexity of linking *in vitro* phenotypes to *in vivo* disease processes.

As 3D *in vitro* systems become more sophisticated, the volume and complexity of data generated by imaging, multi-omics, and drug-response studies increasingly exceed the capacity of standard analytical methods. Computational approaches are therefore essential for experimental design, data processing and interpretation. In this context, artificial intelligence (AI) has emerged as an important driver of innovation in the life sciences, particularly for analyzing large y multidimensional datasets (253, 254). AI methods, including machine learning, deep learning, and natural language processing, can identify patterns, classify biological events, and build predictive models with limited manual input (255). Their integration with 3D *in vitro* systems offers a promising strategy for handling complex, high-throughput datasets and may also help improve organoid technologies, for example by optimizing growth conditions and generating more functional and physiologically relevant models (256–260).

A particularly promising future application of 3D *in vitro* systems is personalized medicine using cells isolated from patients to generate models that can be used to test therapies *in vitro* before treatment, potentially reducing trial-and-error approaches. This strategy is already being explored in colorectal and pancreatic cancer and is likely to expand as the technology becomes more standardized and accessible (261, 262).

3D *in vitro* models are also transforming drug discovery and toxicity testing because they more closely reflect human biology than animal models or 2D cultures, offering the potential for faster development, lower costs, and fewer clinical trial failures. They also provide valuable platforms for studying disease mechanisms and may eventually contribute to regenerative medicine and tissue replacement, although major challenges such as vascularization and immune rejection remain.

In summary, 3D *in vitro* systems are expected to become increasingly important in biomedical research, drug development, and personalized medicine, with longer-term potential in regenerative medicine. Despite current limitations, continued technological and methodological advances are steadily expanding their scope and impact.
